# Psychological Distress After Covid-19 Recovery: Reciprocal Effects With Temperament and Emotional Dysregulation. An Exploratory Study of Patients Over 60 Years of Age Assessed in a Post-acute Care Service

**DOI:** 10.3389/fpsyt.2020.590135

**Published:** 2020-11-12

**Authors:** Delfina Janiri, Georgios D. Kotzalidis, Giulia Giuseppin, Marzia Molinaro, Marco Modica, Silvia Montanari, Beatrice Terenzi, Angelo Carfì, Francesco Landi, Gabriele Sani, The Gemelli Against COVID-19 Post-acute Care Study Group

**Affiliations:** ^1^Department of Psychiatry and Neurology, Sapienza University of Rome, Rome, Italy; ^2^Department of Neuroscience, Section of Psychiatry, Università Cattolica del Sacro Cuore, Rome, Italy; ^3^Department of Psychiatry, Fondazione Policlinico Universitario Agostino Gemelli IRCCS, Rome, Italy; ^4^Department of Neurosciences, Mental Health, and Sensory Organs, Faculty of Medicine and Psychology, Sapienza University of Rome, Rome, Italy; ^5^Geriatrics Department, Fondazione Policlinico Universitario Agostino Gemelli IRCCS, Rome, Italy

**Keywords:** COVID-19, nasopharyngeal swab, nasal swab, emotional dysregulation, affective temperaments, psychological distress, aging

## Abstract

To study the long-term psychological effects of Covid-19 disease, we recruited 61 patients older than 60 years of age and administered the Kessler questionnaire K10 to assess psychological distress and classify them according to mental health risk groups. Patients' affective temperaments were assessed with the 39-item form of the Temperament Evaluation of Memphis, Pisa, Paris, and San Diego (TEMPS-A-39) and emotional dysregulation with the Difficulties in Emotion Regulation Scale (DERS). Patients were divided in two samples according to their scores on the K10, i.e., a high likelihood of psychological distress group (*N* = 18) and a low likelihood of psychological distress group (*N* = 43). The two groups differed on their gender composition, in that more women (*N* = 11) were in the former and more men in the latter (*N* = 29) (χ^2^ = 4.28; *p* = 0.039). The high likelihood of psychological distress group scored higher on the Cyclothymic (3.39 ± 3.45 vs. 0.93 ± 1.08, *p* < 0.001) and the Depressive (2.28 ± 2.82 vs. 0.65 ± 1.09, *p* = 0.01) affective temperaments of the TEMPS and on the lack of Impulse control (12.67 ± 4.04 vs. 9.63 ± 3.14, *p* = 0.003) and lack of Clarity (15.00 ± 5.56 vs. 9.85 ± 4.67, *p* = 0.004) scales of the DERS. Our results show that having had Covid-19 may be related with high likelihood for psychological distress in advanced-age people and this may in turn be associated with impaired emotional regulation and higher scores on depressive and cyclothymic temperaments.

## Introduction

The Covid-19 outbreak and the subsequent lockdown have caused significant distress in the general population in many countries and resulted in various psychological problems in the caregivers (1), healthcare workers (2), and the patients themselves (3–5). Lockdown-related loneliness and isolation may play a part in this distress (6). Personal factors may affect the subsequent development of psychological problems, with people being classified according to their constitution and coping abilities into high-, medium-, and low-risk for the development of psychological symptoms, mainly anxiety, and depression (6). Depressive, anxiety, and sleep symptoms develop in patients with Covid-19 while in the hospital (4), but anxiety may persist after recovery (7).

The response to the Covid-19 pandemic could prove to be analogous to the response to natural disasters or other similar catastrophic events impinging upon a population (8), and may cause permanent distress in the affected population (9). Psychological/psychiatric consequences of disasters may persist as long as 12 years in one out of six members of the affected population (10). The psychological response to the Covid-19 pandemic has been promptly reported; in hardly-hit populations, it is similar to post-traumatic stress disorder (PTSD) symptomatology in the population (11). Similarly, patients who actually developed Covid-19 and survived, are likely to develop PTSD symptomatology (12). Patients with Covid-19 reported many PTSD and depressive symptoms (13). A meta-analysis reported depressed mood, insomnia, anxiety, irritability, memory impairment, fatigue, and traumatic memories as the most frequent complaints in the post-illness stage (14).

While physical symptoms may survive by 3 months the acute Covid-19 phase (15), there is currently a dearth of reports on the long-term psychological response of patients who recovered from Covid-19. Since individual factors determine individual patients' likelihood to develop psychological symptoms (6) and since these affect how each patient deals with life, they may affect coping abilities, and styles and the response to disease. Temperament refers to early-appearing individual differences in emotional reactivity; it is stable across the lifespan and has strong biological underpinnings. It consists of five subtypes, i.e., depressive (dysthymic), cyclothymic, hyperthymic, irritable, and anxious, and is important in determining individual responses to environmental challenge (16). Affective temperament could influence emotion-regulation mechanisms, with particular evidence for the cyclothymic temperament, which has been associated to emotional dysregulation (17). Emotion dysregulation is defined by difficulties in several areas, including the ability to understand and accept emotional experiences, modulate their intensity or duration, and manage emotional reactions in order to meet situational demands and avoid maladaptive behaviors, such as impulsive acts. Emotional dysregulation has been shown to moderate psychological distress (18, 19). Given the intercorrelations between these three constructs, we aimed to assess them through appropriate self-rated instruments in a population of patients who had recovered from Covid-19 and tested negative on two consecutive nasal and/or nasopharyngeal swabs. Our intention was to obtain data that could constitute positive or negative predictors of future psychiatric disorder.

## Materials and Methods

### Patient Sample

Consecutive patients aged >60 years who had contracted Covid-19 infection and recovered were included in this study. Eligible patients were sought from those referring to the multidisciplinary post-acute care service where multiple specialists participate and that has been established at the Fondazione Policlinico Universitario Agostino Gemelli IRCCS, Università Cattolica del Sacro Cuore of Rome (Rome, Italy). (20). Assessment was comprehensive and included medical and psychiatric history, physical examination, and psychiatric status. Clinical characteristics, including clinical and drug treatment history, and other clinical measures, were inserted in a database. All patients were hospitalized at the Fondazione Policlinico Universitario Agostino Gemelli IRCCS and were referred to our post-acute service (Gemelli Against COVID-19 Post-Acute Care Service). Patients, after their discharge had to test negative on two consecutive nasal and/or nasopharyngeal swabs and be afebrile. Patients (*N* = 61) were invited to complete the self-rating questionnaires; they all volunteered. Those unable to provide informed consent or were not sufficiently fluent in Italian to complete the questionnaires were excluded (*N* = 2).

### Psychometric Tools

To assess our sample, we used the following:

#### Difficulties in Emotional Regulation Scale (DERS)

To assess deficits in emotion regulation we used the Difficulties in Emotion Regulation Scale (DERS) (21), a 36-item self-report measure assessing typical levels of emotion dysregulation. Participants are required to rate each item on a 5-point Likert-type scale (1=almost never; 2=sometimes; 3=about half the time; 4=most of the time; and 5=almost always). Items 1, 2, 6, 7, 8, 10, 17, 20, 22, 24, and 34 are scored reverse. The validated Italian version was used (22, 23). The items are distributed on six dimensions: (1) Non-acceptance of emotional responses (NONACCEPT), items 11, 12, 21, 23, 25, and 29; (2) Difficulty engaging in Goal-directed behavior (GOALS), items 13, 18, 20, 26, and 33; (3) Impulse control difficulties (IMPULSE), items 3, 14, 19, 24, 27, and 32; (4) Lack of emotional awareness (AWARENESS), items 2, 6, 8, 10, 17, and 34 (all reverse); (5) Limited access to emotion regulation strategies (STRATEGIES): 15, 16, 22, 28, 30, 31, 35, and 36; and (6) Lack of emotional clarity (CLARITY): 1, 4, 5, 7, and 9. The total score is the sum of all items. Higher scores indicate greater difficulties with regulating emotions. The scale has shown convergent validity with other established measures of emotion dysregulation and fair test-retest reliability, internal consistency, and adequate predictive validity of several behavioral outcomes associated with emotion dysregulation (24, 25). It has no predefined cutoff; each cutoff is tailored to the investigated condition.

#### TEMPS-A-39

We used the validated Italian translation of the shorter, 39-item form of the Temperament Evaluation of Memphis, Pisa, Paris, and San Diego (TEMPS-A-39) (26). This self-rated questionnaire investigates the prevalence of one of the above-mentioned five affective temperaments in an individual; responses in the short version are not as in the full, 110-item version as true or false (27), but rather Yes or No. This instrument has 39 statements with the first 12 referring to the cyclothymic temperament (C), items 13–19 to the depressive (dysthymic) temperament (D), items 20–28 to irritable temperament (I), 29–36 to hyperthymic (H), and 37–39 to the anxious temperament (A). The score on each temperament is the sum of the Yes responses. The tool has obtained evidence of diachronic stability in its various translations (test-retest coefficient range ρ = 0.594–0.84) and good internal consistency (α = 0.682–0.893) (28, 29). The 39-item version has consistently shown a five-factor solution as the best fit (30).

#### K10

We used the K10 [Kessler 10 Psychological Distress Scale; (31)] to assess psychological distress in our post-COVID-19 population. K10, a 10-item questionnaire, provides a global measure of distress experienced in the last 4 weeks. We used the validated Italian translation (32). Each item is scored 1–5 on a Likert scale, where (1) is “None of the time,” (2) “A little of the time,” (3) “Some of the time,” (4) “Most of the time,” and (5) “All of the time;” items 3 and 6 are skipped and rated 1 if the preceding items were scored “None of the time.” Low scores indicate low levels of psychological distress whereas high scores indicate high levels of psychological distress. Consistently with previous validation studies (33, 34), we adopted the cut-off score of >19 to detect the likelihood of presence of psychological distress. The 20 cutoff combined good sensitivity (0.66) and excellent specificity (0.92) in Andrews and Slade (33).

### Study Design

This was a cross-sectional study. After their second consecutive negative nasal or nasopharyngeal swab, patients were invited to complete the three self-rated questionnaires. The testing occurred at the waiting room of the Geriatrics Service of the Columbus post-Covid-Hospital. Specifically-trained psychiatrists were available for psychometric tool application and helped out patients to efficiently complete the questionnaires.

After completing assessments, patients underwent thorough interviews to determine whether they should continue on being seen at the Psychiatric outpatient clinic of the Department of Psychiatry at the the Fondazione Policlinico Universitario Agostino Gemelli IRCCS, Università Cattolica del Sacro Cuore of Rome (Rome, Italy). Special emphasis was placed on their perceived sense of distress and loneliness. Further treatment was agreed upon with treating clinicians according to patient preference.

### Ethics

Each patient was provided with detailed information regarding the purpose and design of the study and was asked to provide written informed consent to participate. We endorsed in this study the Principles of Human Rights, as adopted by the World Medical Association at the 18th WMA General Assembly, Helsinki, Finland, June 1964 and subsequently amended by the 64th WMA General Assembly, Fortaleza, Brazil, October 2013. The study protocol was approved by the Ethics Committee of the Fondazione Policlinico Universitario Agostino Gemelli IRCCS, Università Cattolica del Sacro Cuore of Rome (Rome, Italy). Written informed consent has been obtained from all participants.

### Statistical Analysis

First, we subdivided our sample into two groups according to K10 cutoffs in: (1) subjects without likelihood of psychological distress (total K10 score, <20); (2) subjects with likelihood of psychological distress (total K10 score at least 20). We compared the two groups on socio-demographic and clinical characteristics on the basis of the chi-squared test (χ^2^) for nominal variables and one-way analysis of variance (ANOVA1way).

For the aims of this study, we focused on the distribution patterns of temperament and emotion dysregulation subscales in patents with and without psychological distress. Therefore, we conducted a series of one-way analyses of covariance (ANCOVA), to compare means among groups, setting temperament and emotion dysregulation subscales as dependent variables. Age, Sex, Living alone, Length of hospitalization, Admission to ICU, Use of Immunomodulating therapies, and Post-hospitalization interval until the assessment were inserted as covariates to control the statistical model for these variables. We used the statistical routines of SPSS Statistics 24.0 for Windows (IBMCo., Armonk, NewYork, United States, 2016).

### Results

In our sample (*n* = 61), 18 subjects (29.51%) reported psychological distress. Sociodemographic and clinical characteristics are summarized in Table 1. The only significant difference was that there were more women than men in the group with likelihood of psychological distress (*N* = 11, 61.11% women vs. *N* = 7, 38.89% men) and less women than men in the group without likelihood of psychological distress (*N* = 14, 32.56% women vs. *N* = 29, 67.44% men) (χ^2^ = 4.28; *p* = 0.039). The two groups did not differ in other sociodemographic characteristics as for, living alone, lifetime history of psychiatric disorders, and COVID-19 related clinical characteristic (Length of hospitalization, Admission to Intensive care unit, Use of Immunomodulating therapies, and Post-hospitalization interval until the assessment).

**Table 1:** Sociodemographic and clinical characteristics.

**Characteristics**	**No psychological distress (K10 <20)**	**Psychological distress (K10≥20)**	**χ^**2**^ or F**	**df**	***p***
Overall sample, *n* (%)	43 (70.49)	18 (29.51)			
Females, *n* (%)	14 (32.56)	11 (61.11)	4.28	1	0.039^*^
Age (Y), mean ± SD	67.98 ± 6.52	65.61 ± 6.25	1.71	1	0.196
Educational level (Y), mean ± SD	14.40 ± 4.60	11.64 ± 4.80	3.51	1	0.067
Occupational status, *n* (%)			5.31	2	0.070
Employed	15 (34.9)	7 (38.9)			
Unemployed	0 (0.0)	2 (11.1)			
Retired	28 (65.1)	9 (50.0)			
Marital status, *n* (%)			2.74	1	0.098
Married/living with partner	31 (72.1)	9 (50.0)			
Unmarried, living alone	12 (27.9)	9 (50.0)			
Ling alone, *n* (%)	8 (18.6)	3 (16.7)	0.03	1	0.85
Lifetime history of psychiatric disorders, *n* (%)	9 (20.9)	8 (44.4)	3.49	1	0.062
Length of hospitalization (Days), mean ± SD	15.36 (9.67)	19.50 (12.35)	1.92		0.71
Admission to ICU, *n* (%)	5 (11.6)	4 (22.2)	1.13	1	0.28
Use of Immunomodulating therapies, *n* (%)	15 (37.5)	8 (57.1)	1.63	1	0.20
Post-hospitalization interval (Days), mean ± SD	40.69 (18.87)	40.55 (18.67)	0.001	1	0.97

* *p < 0.05; Significant results in bold characters. df, degrees of freedom; F, value of variance of the group means; M, mean; p, statistical significance; SD, standard deviation; Y, years; χ^2^, chi-squared test, ICU, intensive care unit*.

A series of ANCOVAs showed that the group with psychological distress reported significantly higher scores on the cyclothymic (*p* < 0.001) and depressive temperaments (*p* = 0.01) than the one without psychological distress (Table 2). The high likelihood for psychological distress group also reported more impulsivity (*p* = 0.003) and lack of emotional clarity (*p* = 0.004) than individuals without likelihood of psychological distress (Table 2).

**Table 2:** Psychometric characteristics.

**Characteristics**	**No psychological distress [N = 43]**	**Psychological distress [N = 18]**	**χ^2^ or F**	**df**	***p***
**Temperament evaluation of Memphis, Pisa, Paris and San Diego autoquestionnaire (TEMPS-A)**					
TEMPS-A cyclothymic, $\bar{x}$ ± SD	0.93 ± 1.08	3.39 ± 3.45	15.29	1	<0.001^***^
TEMPS-A depressive, $\bar{x}$ ± SD	0.65 ± 1.09	2.28 ± 2.82	6.83	1	0.01^*^
TEMPS-A irritable, $\bar{x}$ ± SD	0.61 ± 1.02	1.17 ± 1.34	2.74	1	0.10
TEMPS-A hyperthymic, $\bar{x}$ ± SD	4.54 ± 1.96	4.61 ± 2.79	0.21	1	0.64
TEMPS-A anxious, $\bar{x}$ ± SD	0.93 ± 0.90	1.22 ± 0.88	0.003	1	0.94
**Difficulties in emotion regulation scale (DERS)**					
DERS Non-acceptance, $\bar{x}$ ± SD	11.27 ± 5.08	12.78 ± 5.33	0.76	1	0.38
DERS Goals, $\bar{x}$ ± SD	11.05 ± 4.25	12.28 ± 3.95	0.12	1	0.72
DERS Impulse, $\bar{x}$ ± SD	9.63 ± 3.14	12.67 ± 4.04	9.79	1	0.003^**^
DERS Awareness, $\bar{x}$ ± SD	16.15 ± 5.41	16.28 ± 6.56	0.00	1	0.98
DERS Strategies, $\bar{x}$ ± SD	13.73 ± 4.49	15.61 ± 5.36	0.90	1	0.34
DERS Clarity, $\bar{x}$ ± SD	9.85 ± 4.67	15.00 ± 5.56	9.23	1	0.004^**^

* p < 0.05;

** p < 0.01;

*** *p < 0.001. Significant results in bold characters. Abbreviations: DERS, Difficulties in Emotion Regulation Scale; df, degrees of freedom; F, value of variance of the group means; p, statistical significance; SD, standard deviation; TEMPS-A, Temperament Evaluation of Memphis, Pisa, Paris and San Diego Autoquestionnaire; $\bar{x}$, mean; χ^2^, chi-squared test. Model controlled for Age, Sex, Living alone, Length of hospitalization, Admission to ICU, Use of Immunomodulating therapies, and Post-hospitalization interval*.

### Discussion

In this study we found people who fully recovered from Covid-19 and who display at least two consecutive negative nasal/nasopharyngeal swabs to show considerably more psychological distress, as measured through the K10, than the Italian and worldwide general population (32, 35). We also found Post-Covid-19 women to be more vulnerable to psychological distress than their male counterparts. Patients who recovered from Covid-19 and who reported psychological distress presented with more occurrences of cyclothymic and depressive affective temperaments and scored higher on the DERS scale dimensions of lack of impulse control and lack of clarity.

In our study we found 29.51% of our sample to have high psychological distress. This prevalence is high for an advanced-age population (35). A previous study found only 1% of elderly Canadians to score above 15 on the K10, with an optimum cutoff for mild depressive symptoms to be in the 20–23 range after receiver operator characteristics (ROC) analysis (36). The fact that women are more vulnerable to psychological distress is in line with what is found in literature for both Covid-19 and other patient populations. Women generally report higher degrees of psychological distress (37, 38). This holds true also for the Covid-19 threat in the general population (39–41).

Our study showed cyclothymic and depressive temperaments to constitute predictors of psychological distress in patients who recovered from Covid-19. Depressive temperament is characterized by pessimism, high self-criticism, and affective dependency, whereas cyclothymic temperament is marked by sudden shifts in mood, energy, behavior, and thinking. Our results match those of a recent study investigating the psychological distress perceived by the Italian general population during the early phase of the COVID-19 pandemic (41). This study found cyclothymic, depressive, and anxious temperaments, along with adult attachment styles, to be specific risk factors for psychological distress. In particular, they found the insecure-anxious attachment dimension “Need for approval” of the Attachment Style Questionnaire (ASQ) to constitute a risk factor, while the ASQ “Confidence” and “Discomfort with closeness” dimensions of the secure and avoidant attachment styles to be protective from psychological distress. They hypothesized that cyclothymic/depressive individuals would be more likely to perceive the COVID-19 outbreak and the related social isolation as distressful and to experience increased negative affect in response to the pandemic (41). Our results suggest that this can be extended to patients who recovered from Covid-19. Data match those of another study conducted before Covid-19, which showed that students with high distress scored higher on the cyclothymic, depressive, irritable, and anxious TEMPS temperaments, compared to those with low psychological distress (19); in this study the authors assessed psychological distress trough the 12-item General Health Questionnaire (GHQ-12), in contrast to us, who used the K10. However, the two instruments have shown similar psychometric properties, internal consistency, and convergent validity (42), although the K10 performed slightly better than the GHQ-12 in one study (43) and identified more cases in another (44).

Our study highlights that emotional dysregulation could mediate the development of psychological distress in patients who recovered from Covid-19. Accordingly, deficits in affect regulation have also been observed in healthy individuals at risk for psychopathology and could influence the development of psychiatric symptoms in the context of stressful events (45). Nevertheless, the specific relationship between psychological distress and emotional dysregulation has been little investigated in literature. Psychological distress was shown to correlate with all DERS dimensions, save for Awareness, in a sample of university students of medium proportions (46) and with the Strategies, Impulse, and Clarity subscales in a small sample of patients with alcohol use disorder (47). Nevertheless, these data are not fully comparable with ours, since despite using the DERS, both these studies differed in the instrument used to assess psychological distress and none used the K10.

Our findings indicate that, among DERS dimensions, the lack of impulse control and clarity, along with with depressive and cyclothymic temperaments were associated with post Covid-19–related psychological distress. Interestingly, the lack of impulse control has been linked with the instability of cyclothymia (48). This is probably caused by reduced impulse control when mood is high and heightened reactions to experiences that are perceived as pleasurable. The lack of clarity about the nature of one's own emotions could also be linked with the tendency toward shifts in mood and energy. Furthermore, the cognitive uncertainty characterizing depressive traits could also include difficulties in recognizing emotional responses. In agreement with this, a specific correlation was found between the depressive and cyclothymic TEMPS temperament and DERS Impulse and Clarity scores (49).

In our advanced-age patients with past Covid-19 infection, who successfully recovered and were asymptomatic, we found no effect of loneliness on psychological distress, as measured through their marital/partnership status. This is not consistent with the finding that living alone was an independent predictor of psychological distress in an aged sample of healthy individuals (50). This result could be potentially explained by the effect of Covid-19–related forced isolation, which might overcome the effect of loneliness on psychological distress.

Taken together our data suggest that the past Covid-19 experience has enduring effects that affect psychological well-being and psychological distress; in turn, this exposes the individual to the likelihood of mental disease, especially anxiety and depressive disorders (31, 34, 36). An assessment of post-disaster disorders, like posttraumatic stress disorder, is mandatory. In fact, this disorder shares many clinical features with the above disorders, and patients with it are likely to score high on the K10 (51). The prompt response of mental health services to these new requirements could avoid the development of full-blown psychiatric disorders and ease public burden. Services could provide programs similar to those enforced or proposed for other PTSD-stricken populations (52, 53).

#### Limitations

This study has several limitations. First, its cross-sectional design prevents us from drawing conclusions on the causal relationships of the post-Covid-19 state and temperament, psychological distress, and difficulties in emotional regulation. Second, the small sample size may have limited the power of the study; hence, these findings should be intended as exploratory. The small convenience sample was due to the very specific population we wanted to assess (Consecutive patients aged >60 years who had contracted Covid-19 infection and recovered). Future studies with larger sample size are needed to confirm our initial speculations. Third, we specifically aimed to investigate whether Covid-19 has a long-term impact on psychological health in elderly people, and obtained evidence that it increases the likelihood of belonging to a high psychological distress group. These observations should be replicated in post-Covid-19 patients of other age ranges as well. Fourth, we included only patients who were hospitalized at the Fondazione Policlinico Universitario Agostino Gemelli IRCCS in Rome, Italy, and who were referred to the multi-specialized Gemelli Against COVID-19 Post-Acute Care. There are very few hospitals in Italy offering this type of service, preventing us from currently generalizing our results to other populations. Finally, the lack of information on previous history of personal distress is another limitation of our study. This is a potential shortcoming because past adverse events are specific risk factors for psychiatric symptoms (54, 55) and may increase vulnerability to the stressful effect of COVID-19 outbreak. Despite limitations, this is one of the few studies presenting data on patients recovered from the Covid-19 illness, assessing in person patients and finding a specific link between psychological distress and personality characteristics.

### Conclusions

In this study we tested psychological constructs like psychological distress, difficulty with regulating emotions, and affective temperament dimensions in people who recovered from Covid-19 after their nasal or nasopharyngeal swabs were negative at least twice. We found the high likelihood for psychological distress group to score higher on the depressive (dysthymic) and cyclothymic affective temperaments and on the Impulsivity and (lack of) Clarity scales of the Difficulties in Emotion Regulation Scale. This population is worth investigating with other measures as well, using greater samples and longitudinal designs.

## Data Availability Statement

The raw data supporting the conclusions of this article will be made available by the authors, without undue reservation.

## Ethics Statement

The studies involving human participants were reviewed and approved by Ethics Committee of the Fondazione Policlinico Universitario Agostino Gemelli IRCCS, Università Cattolica del Sacro Cuore of Rome. The patients/participants provided their written informed consent to participate in this study.

## Author Contributions

DJ, GDK, GG, MMol, MMod, SM, BT, AC, FL, and GS wrote the paper. All authors read and approved the final draft.

## The Gemelli Against COVID-19 Post-acute Care Study Group

### Steering Committee

Francesco Landi, Elisa Gremese.

### Coordination

Roberto Bernabei, Massimo Fantoni, Antonio Gasbarrini.

### Field Investigators

Carlo Romano Settanni, Francesca Benvenuto, Giulia Bramato, Vincenzo Brandi, Angelo Carfì, Francesca Ciciarello, M. Rita Lo Monaco, Anna Maria Martone, Emanuele Marzetti, Carmen Napolitano, Francesco Cosimo Pagano, Sara Rocchi, Elisabetta Rota, Andrea Salerno, Matteo Tosato, Marcello Tritto, Riccardo Calvani, Lucio Catalano, Anna Picca, Giulia Savera, Roberto Cauda, Enrica Tamburrini, Alberto Borghetti, Simona Di Gianbenedetto, Rita Murri, Antonella Cingolani, Giulio Ventura, Eleonora Taddei, Davide Moschese, Arturo Ciccullo, Leonardo Stella, Giovanni Addolorato, Francesco Franceschi, Geltrude Mingrone, Maria Assunta Zocco, Maurizio Sanguinetti, Paola Cattani, Simona Marchetti, Brunella Posteraro, Michela Sali, Alessandra Bizzarro, Alessandra Lauria, Stanislao Rizzo, Maria Cristina Savastano, Gloria Gambini, Grazia Maria Cozzupoli, Carola Culiersi, Giulio Cesare Passali, Gaetano Paludetti, Jacopo Galli, Lucia D'Alatri, Fabrizio Crudo, Giovanni Di Cintio, Ylenia Longobardi, Laura Tricarico, Mariaconsiglia Santantonio, Danilo Buonsenso, Piero Valentini, Davide Pata, Dario Sinatti, Cristina De Rose, Luca Richeldi, Francesco Lombardi, Angelo Calabrese, Gabriele Sani, Delfina Janiri, Giulia Giuseppin, Marzia Molinaro, Marco Modica, Luigi Natale, Anna Rita Larici, Riccardo Marano, Annamaria Paglionico, Luca Petricca, Laura Gigante, Gerlando Natalello, Anna Laura Fedele, Marco Maria Lizzio, Barbara Tolusso, Stefano Alivernini, Angelo Santoliquido, Luca Santoro, Antonio Nesci, Valentina Popolla, Giorgia Mari, Raffaella Marchese, Carolina Ausili Cefaro.

## Conflict of Interest

The authors declare that the research was conducted in the absence of any commercial or financial relationships that could be construed as a potential conflict of interest.
